# Global research hotspots and trends in oxidative stress-related diabetic nephropathy: a bibliometric study

**DOI:** 10.3389/fendo.2024.1451954

**Published:** 2025-01-10

**Authors:** Xiao-rong Wang, Zeng Wu, Tong-tong He, Xiao-han Chen, Xiao-fei Jin, Chun-yue Zuo, Shao-ze Yang, Yu Gao, Xiao-hong Zhou, Wei-juan Gao

**Affiliations:** Hebei Key Laboratory of Chinese Medicine Research on Cardio-Cerebrovascular Disease, Hebei University of Chinese Medicine, Shijiazhuang, China

**Keywords:** diabetic nephropathy, oxidative stress, VOSviewer, Bibliometrix R package, CiteSpace

## Abstract

**Background:**

Oxidative stress is widely acknowledged as a key pathogenic mechanism in diabetic nephropathy (DN). In recent years, the role of oxidative stress in DN has garnered increasing attention. However, no bibliometric analysis has yet been conducted on the relationship between oxidative stress and DN. This study aims to systematically analyze the relevant literature, identify trends in research, assess current hotspots, and predict future directions.

**Methods:**

We retrieved literature related to oxidative stress and DN from the Web of Science Core Collection database. We analyzed data on publication volume, countries/regions, institutions, journals, keywords, and other relevant metrics using VOSviewer, the Bibliometrix R package, and CiteSpace.

**Results:**

From 2014 to 2024, a total of 4076 publications related to oxidative stress and DN were published across 755 journals, showing a consistent upward trend each year. China and the United States are the leading contributors in this field and demonstrate close collaborative efforts. The top contributors by country, institution, journal, and author include: China (1919 publications), Jilin University and Central South University (69 publications each), *BIOMEDICINE & PHARMACOTHERAPY* (117 publications), and Prof. Sun Lin (33 publications). The most frequent keyword is “oxidative stress” (3683 occurrences). In the co-citation analysis, Alicic RZ’s 2017 study was the most cited (144 citations). These findings highlight the critical importance of investigating the pathogenesis of DN from the oxidative stress perspective.

**Conclusion:**

This study demonstrates a steady increase in research on oxidative stress in DN since 2014, highlighting its central role in the pathogenesis of DN. Future research should focus on the molecular mechanisms of oxidative stress in DN and explore its therapeutic potential, to provide new strategies for the prevention and treatment of DN.

## 1 Introduction

Diabetes mellitus (DM) is a global public health concern, affecting millions of individuals worldwide. In 2021, global healthcare expenditures related to diabetes amounted to approximately USD 966 billion, with projections indicating a rise to USD 1.045 trillion by 2045 (1). DM leads to various vascular complications, including diabetic nephropathy (DN), diabetic retinopathy (DR), neuropathy, and cardiovascular diseases (CVD) (2). DN is one of the most common and severe complications of DM (3), and it is a leading cause of end-stage renal disease (ESRD) (4). Approximately one-third of DM patients progress to DN, and its prevalence has been rising alarmingly (5). The clinical manifestations of DN primarily include hypertension, edema, and proteinuria, which may progress to renal failure or even life-threatening conditions (6).

The pathogenesis of DN involves several interconnected processes, including oxidative stress, activation of the renin-angiotensin-aldosterone system, and inflammatory responses (7–9). Numerous studies have identified oxidative stress as one of the primary mechanisms underlying DN (10, 11), with prolonged hyperglycemia acting as a trigger for its onset (12). Once oxidative stress occurs, the balance between oxidants and antioxidants is disrupted (13). Excessive production of reactive oxygen species (ROS) and reactive nitrogen species (RNS) leads to renal damage, particularly by ROS, which include peroxides, superoxides, and hydroxyl radicals—key contributors to kidney disease (14, 15). Antioxidants, which can be classified as either enzymatic or non-enzymatic, neutralize ROS to maintain cellular homeostasis (16). Excessive ROS production and collapse of the antioxidant system lead to the continuous oxidation of macromolecules, including lipids, proteins, and DNA, ultimately causing cellular dysfunction (8, 17). Research has shown that ROS-induced damage to glomerular endothelial cells, mesangial cells, podocytes, and renal tubular epithelial cells has accelerated the progression of DN (18–20). Consequently, oxidative stress is intricately linked to DN, and recent research has increasingly focused on the potential of medicinal plants and natural compounds in mitigating oxidative stress (21). Understanding the underlying mechanisms of oxidative stress is, therefore, crucial for preventing DN and developing new therapeutic strategies.

Bibliometrics is a statistical method used to analyze large volumes of content or citations (22, 23), providing a quantitative description of existing literature (24). This approach enables researchers to gain insights into key authors, keywords, journals, and countries/regions within a specific research field, thereby facilitating an understanding of thematic evolution and emerging research trends (25, 26). To date, no bibliometric study has been conducted specifically on oxidative stress in the context of DN. This study aims to fill this gap.

Despite significant progress in research on oxidative stress mechanisms in DN, there is a lack of bibliometric studies specifically focusing on oxidative stress in DN. The primary objective of this bibliometric study is to systematically analyze the research landscape, identify hotspots, and explore emerging trends in the field of oxidative stress DN. By offering a comprehensive overview of key contributors and research dynamics, this study seeks to assist basic scientists in prioritizing research directions, facilitating interdisciplinary collaborations, and addressing under-investigated areas. The findings also offer critical insights to advance therapeutic strategies, foster translational applications, and inform resource allocation and policy-making in DN management.

## 2 Materials and methods

### 2.1 Search strategy

Literature data were independently retrieved, validated, and standardized by the authors (WXR) and (WZ) through the Web of Science Core Collection (WoSCC) database, utilizing the Science Citation Index Expanded. The search formula used was as follows:

Ts=(“diabetic nephropathy” OR “diabetic kidney disease”) AND Ts=(“oxidative stress” OR “ROS” OR “reactive oxygen species”).The language was set to English, and only original articles and reviews that met the criteria for this study were included. The search period was from January 1, 2014, to November 1, 2024. Retracted articles or those irrelevant to the topic were manually excluded. The online literature, including full records and references, was then exported in plain text format.

### 2.2 Data analysis

GraphPad Prism 10.1.1 was used to generate line graphs illustrating annual publication trends.

VOSviewer 1.6.20 is a tool designed to construct and visualize bibliometric maps. It creates maps of authors or journals based on co-citation data (27). In this study, VOSviewer was utilized to visualize collaborations among countries/regions, journals, institutions, and authors and to generate network visualizations.

CiteSpace 6.3.R1 (Drexel University, Philadelphia, USA) is a Java-based software developed by Professor Chaomei Chen. Its primary advantage is its ability to explore quantitative data and development trends in scientific research fields through visual analysis, enhancing the understanding of scientific progress (26, 28). In this study, CiteSpace was used to process the literature data collected from WoSCC, constructing visual networks of keywords and references. Additionally, burst detection, cluster analysis of co-cited references, and timeline visualizations were performed. Each node represents a unique keyword or reference, with the size of the node corresponding to its frequency of occurrence; larger nodes indicate higher frequency.

Bibliometrix, an R package developed by Massimo Aria and Corrado Cuccurullo for data analysis and visualization (29), was used in this study to analyze the country affiliations of corresponding authors and visualize the international collaboration network and journal data.

## 3 Results

### 3.1 Publication trends

**Figure 1:**
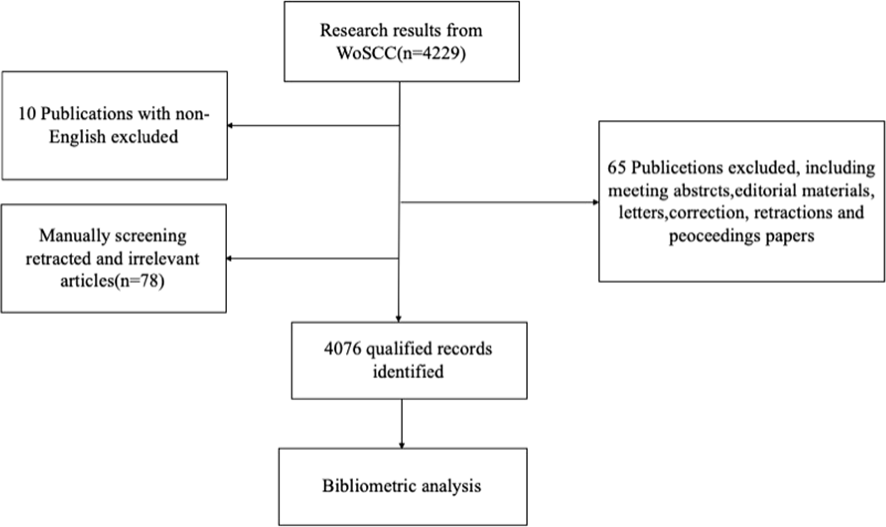
Retrieval process flowchart for the research.

**Figure 2:**
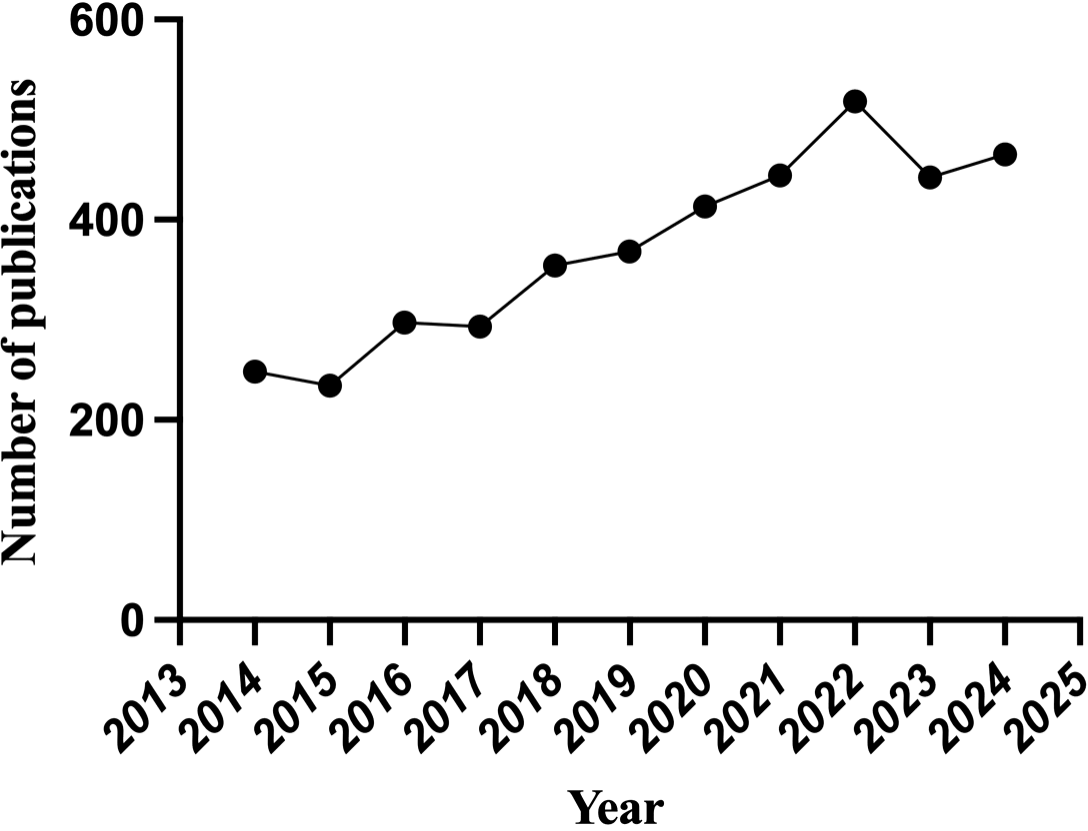
Annual publication trends.

A total of 4229 publications related to oxidative stress and DN were identified from 2014 to 2024.After excluding non-original articles and reviews (n=65), non-English articles (n=10), and manually screening 78 retracted or irrelevant publications, a final total of 4076 publications were retained (**Figure 1**). Since 2014, the number of publications has shown an overall upward trend, with declines observed only in 2015, 2017, and 2023 compared to the previous year. On average, 408 publications were published annually, with the highest number of publications occurring in 2022, which reached 518 articles (**Figure 2**).

### 3.2 Countries/regions and institutions distribution

#### 3.2.1 Countries/regions

**Figure 3:**
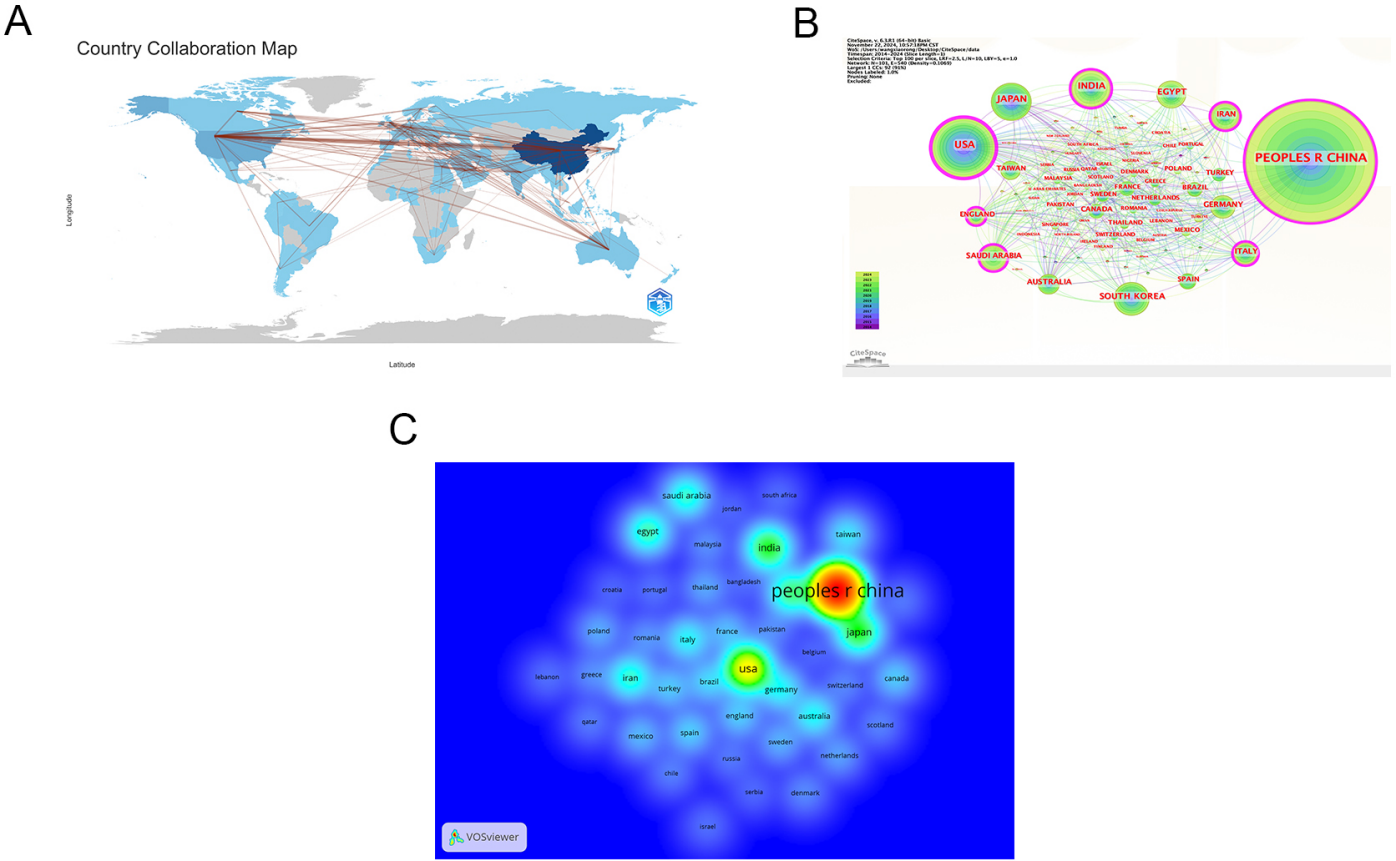
Countries/regions cooperation network. **(A)** Map of cooperation networks across countries/regions; **(B)** Map of central national/regional cooperation network; **(C)** Density map of national/regional cooperation network. Note:the Taiwan in the figure is China’s Taiwan Province. From: CiteSpace.

The 4076 publications were contributed by 3728 institutions across 101 countries/regions. As shown in **Table 1**, the country with the highest contribution is China (n=2025), followed by the United States (n=498), Japan (n=247), India (n=246), South Korea (n=184), Egypt (n=151), Saudi Arabia (n=132), Iran (n=132), Australia (n=102), and Italy (n=97). The top ten countries are distributed across Asia (n=6), Europe (n=1), Oceania (n=1), the Americas (n=1), and Africa (n=1). **Figure 3A** illustrates the collaborative relationships between countries/regions in this field. As shown in **Figure 3B**, each node represents a country or region, with node size indicating centrality, and the color of the circles representing different years. Both China and the United States occupy the most central positions. **Figure 3C** shows a density heatmap of countries/regions generated using VOSviewer, where darker colors indicate closer collaboration.

**Table 1:** Top 10 countries with the most publications.

Rank	Countries	Publications	Link Strength
1	China (Asia)	2025	282
2	USA (America)	498	399
3	Japan (Asia)	247	81
4	India (Asia)	246	96
5	South Korea (Asia)	184	67
6	Egypt (Africa)	151	114
7	Saudi Arabia (Asia)	132	149
8	Iran (Asia)	132	65
9	Australia (Oceania)	102	117
10	Italy (Europe)	97	75

#### 3.2.2 Institutions

**Figure 4:**
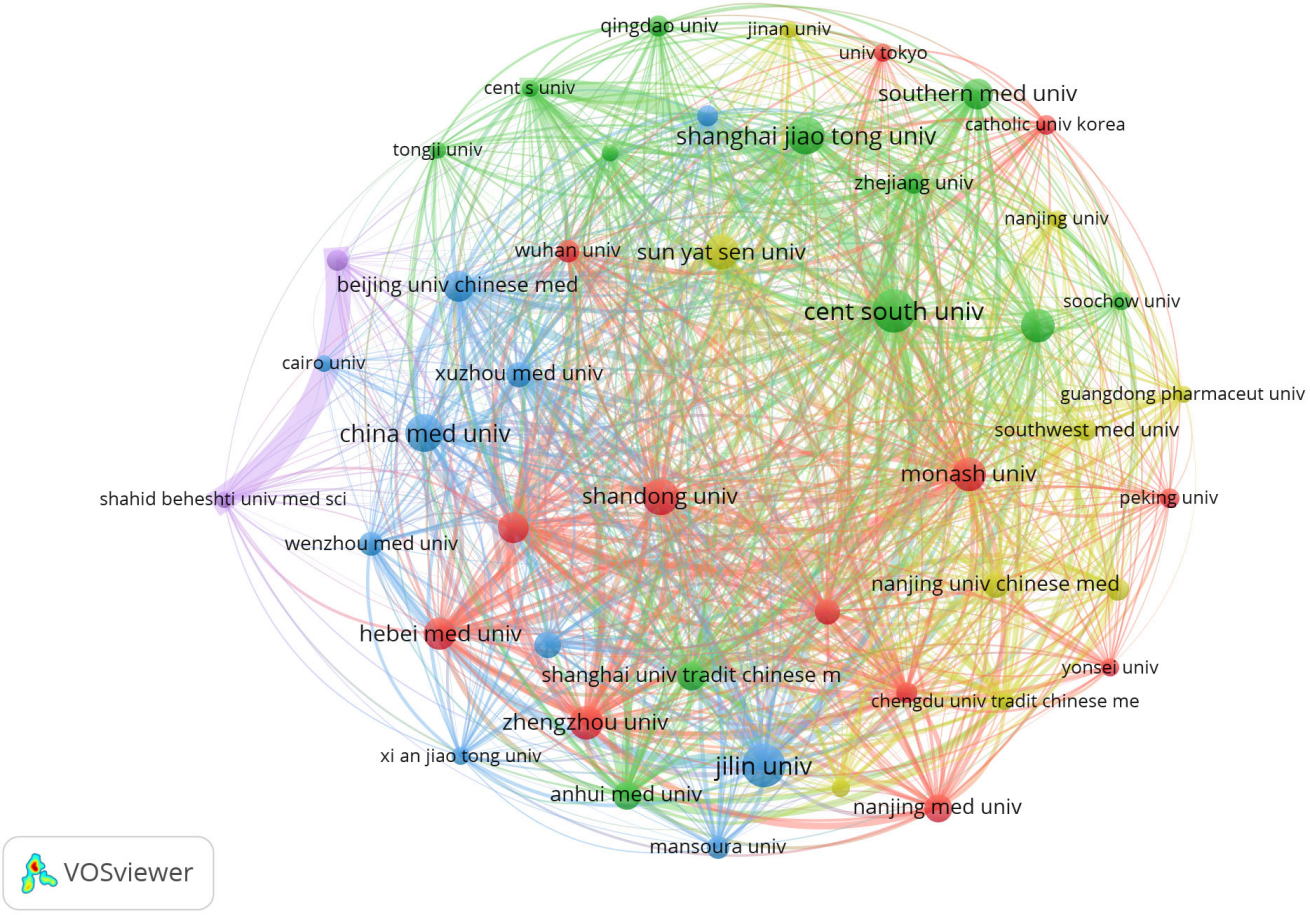
Cooperation network between major institutions. From: VOSviewer.

Regarding institutions, a total of 3728 institutions contributed to the publications, with the top contributors being Jilin University (n=69) and Central South University (n=69), followed by China Medical University (n=61), Shanghai Jiao Tong University (n=58), and Shandong University (n=57), among others, as shown in **Table 2**. Notably, nine out of the top ten institutions by publication count are located in China. The visualization network of institutional collaborations (**Figure 4**) demonstrates that institutions within the same country tend to exhibit closer collaborative ties.

**Table 2:** Top 10 institutions contributing to number of articles.

Rank	Institutions	Publications	Link Strength
1	Jilin University (China)	69	40
2	Cent South University (China)	69	18
3	China med University (China)	61	10
4	Shanghai Jiao Tong University (China)	58	47
5	Shandong University (China)	57	24
6	Sun Yat Sen University (China)	56	46
7	Zhengzhou University (China)	54	12
8	Fudan University (China)	52	38
9	Monsh University (Australia)	52	15
10	Hebei med University (China)	48	6

### 3.3 Journals and co-cited journals

#### 3.3.1 Journals

**Figure 5:**
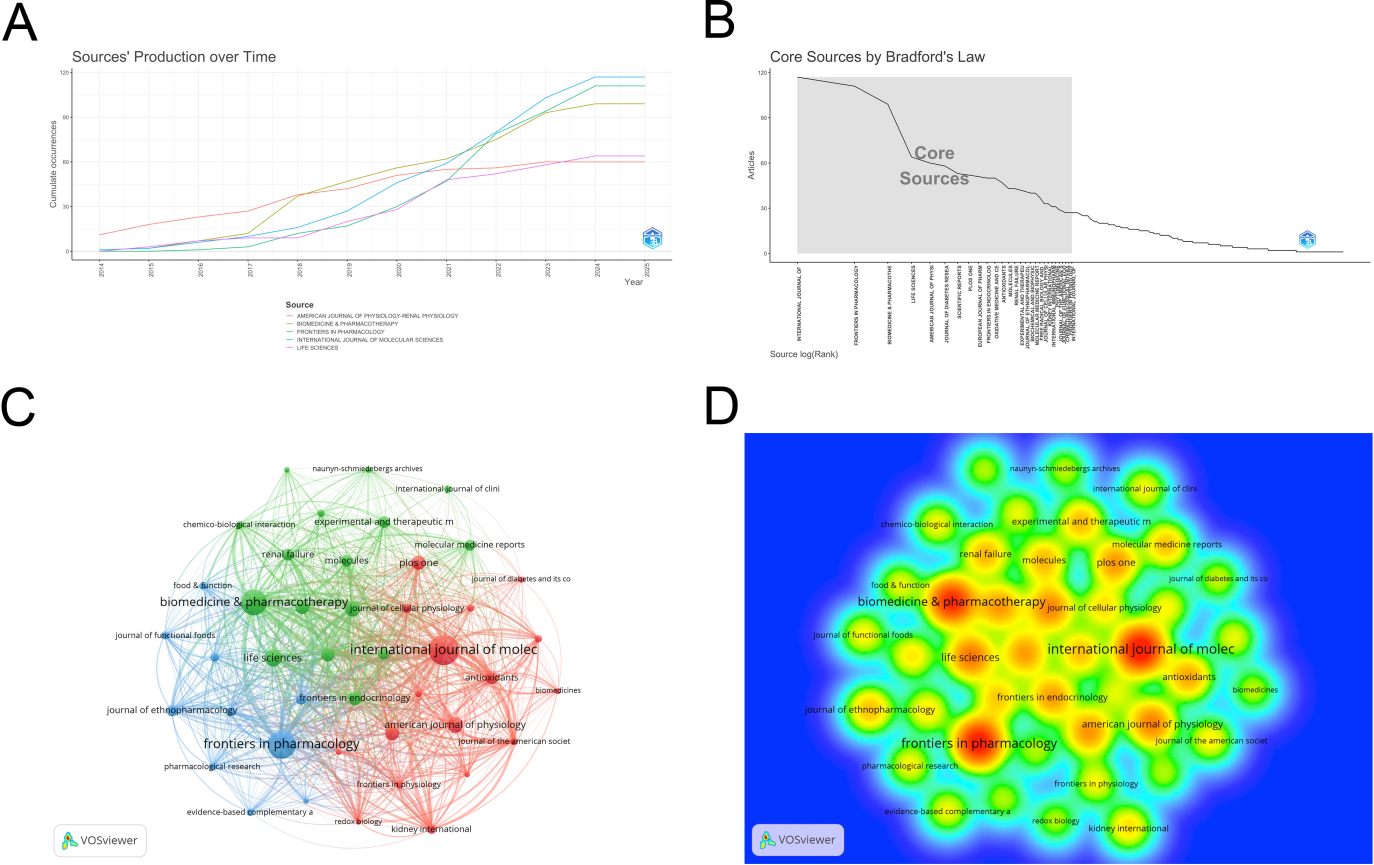
**(A)** Time chart of publications across five journals; **(B)** Core journal collection; **(C)** Journal cooperation network map; **(D)** Density map of journal cooperation network. From: VOSviewer.

The 4076 publications appeared in 755 journals. The top ten journals by publication count are listed in **Table 3**: *INTERNATIONAL JOURNAL OF MOLECULAR SCIENCES* (n=117), *FRONTIERS IN PHARMACOLOGY* (n=111), *BIOMEDICINE* & *PHARMACOTHERAPY* (n=99), *LIFE SCIENCES* (n=64), *AMERICAN JOURNAL OF PHYSIOLOGY-RENAL PHYSIOLOGY* (n=60), *JOURNAL OF DIABETES RESEARCH* (n=58), *SCIENTIFIC REPORTS* (n=53), *PLOS ONE* (n=52), *EUROPEAN JOURNAL OF PHARMACOLOGY* (n=51), and *FRONTIERS IN ENDOCRINOLOGY* (n=50). The highest impact factor among the top ten journals is found in *BIOMEDICINE* & *PHARMACOTHERAPY* (IF=6.9), while the lowest is in *PLOS ONE* (IF=2.9). The average impact factor is 5.4 (obtained from the 2024 Journal Citation Report), indicating that the articles published in these journals are generally of high quality. **Figure 5A** shows the publication timeline of the top five journals. **Figure 5B** illustrates the 28 core journals in this field. VOSviewer was used to filter journals with more than 20 publications, resulting in 43 journals, and a collaboration network between these journals was visualized (**Figure 5C**). **Figure 5D** shows the density heatmap of these journals, where darker colors indicate stronger collaboration.

**Table 3:** Top 10 journals and co-cited journals with the highest number of articles.

Rank	Journal	Frequency	IF	Cited Journal	Frequency	IF
1	INTERNATIONAL JOURNAL OF MOLECULAR SCIENCES	117	4.9	KIDNEY INT	9158	14.8
2	FRONTIERS IN PHARMACOLOGY	111	4.4	J AM SOC NEPHROL	8810	10.3
3	BIOMEDICINE & PHARMACOTHERAPY	99	6.9	DIABETES	7486	6.2
4	LIFE SCIENCES	64	5.2	AM J PHYSIOL-RENAL	5756	3.7
5	AMERICAN JOURNAL OF PHYSIOLOGY-RENAL PHYSIOLOGY	60	3.7	J BIOL CHEM	4538	4.0
6	JOURNAL OF DIABETES RESEARCH	58	3.6	PLOS ONE	4301	2.9
7	SCIENTIFIC REPORTS	53	3.8	J CLIN INVEST	3207	13.3
8	PLOS ONE	52	2.9	DIABETOLOGIA	3105	8.4
9	EUROPEAN JOURNAL OF PHARMACOLOGY	51	4.2	DIABETES CARE	3080	14.8
10	FRONTIERS IN ENDOCRINOLOGY	50	3.9	NEW ENGL J MED	2941	96.2

#### 3.3.2 Co-cited journals

**Figure 6:**
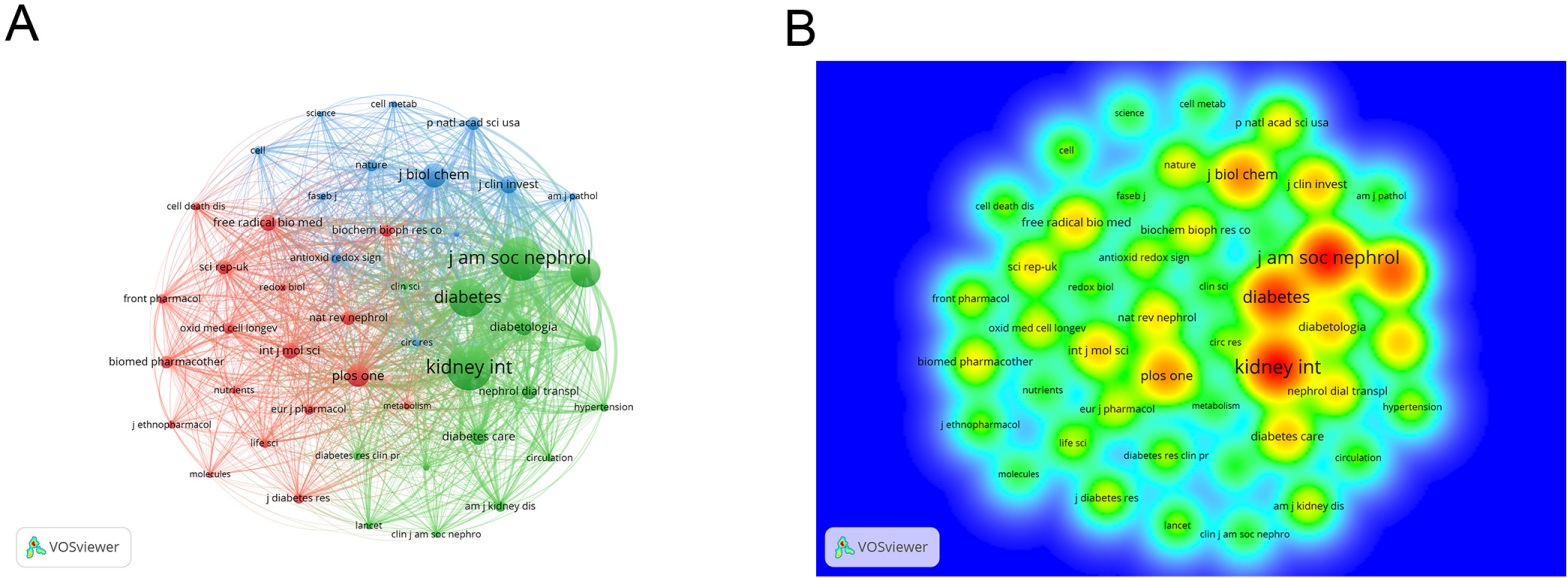
**(A)** Co-cited journal cooperation network map; **(B)** Density map of co-cited journal cooperation network. From: VOSviewer.

In the co-citation analysis, 10,908 co-cited journals were identified. As shown in **Table 3**, the most co-cited journal is *KIDNEY INTERNATIONAL* (n=9158). The highest impact factor among the top ten co-cited journals is *NEW ENGLAND JOURNAL OF MEDICINE* (IF=96.2), while the lowest is *PLOS ONE* (IF=2.9), with an average impact factor of 17.5, indicating that the co-cited journals are generally of high quality. The collaboration network of co-cited journals is shown in **Figure 6A**, where many journals exhibit collaborative links. **Figure 6B** displays the heatmap of co-cited journals, with higher intensity colors representing more frequent collaborations.

### 3.4 Authors and co-cited authors

#### 3.4.1 Authors

**Figure 7:**
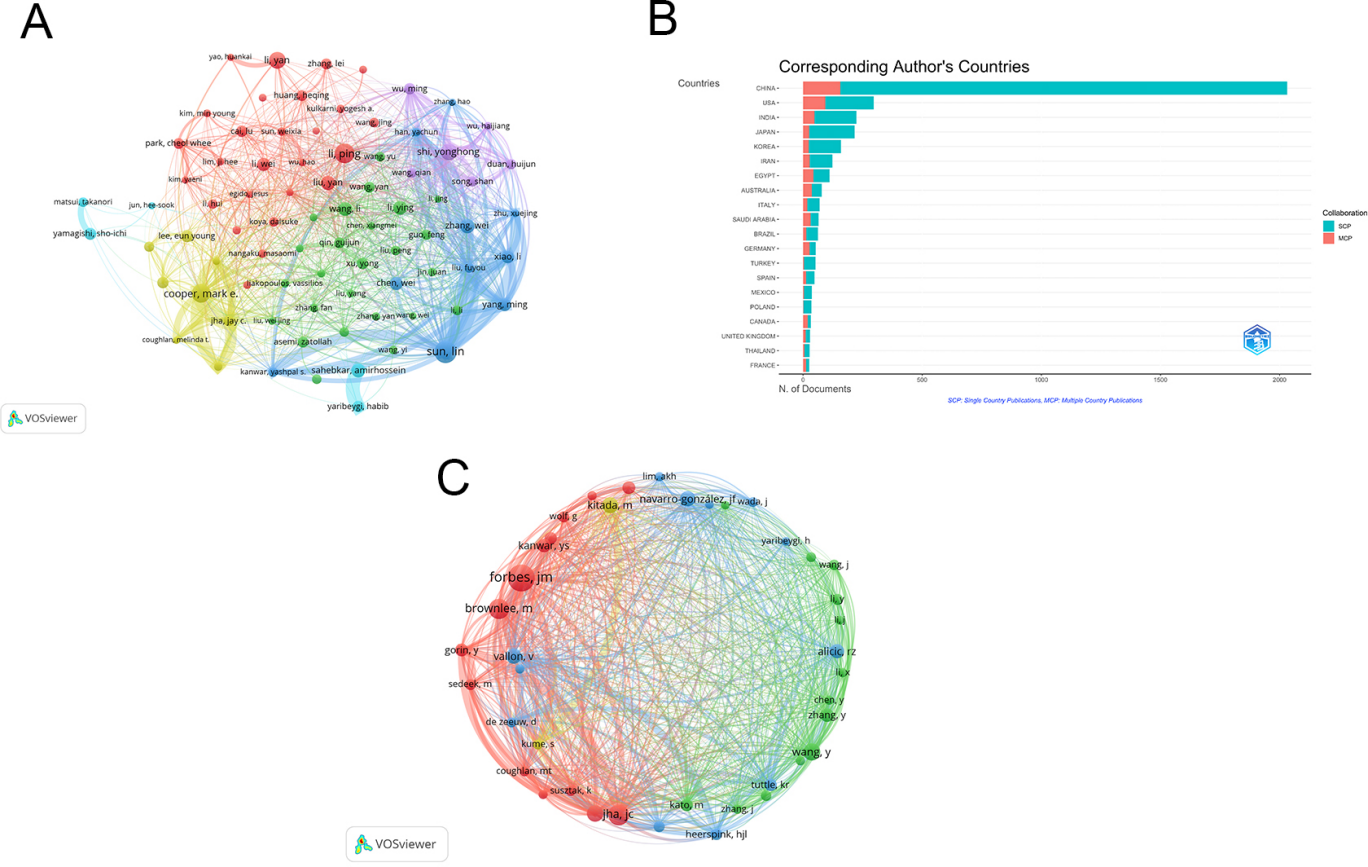
**(A)** Authors cooperation network map; **(B)** Countries of corresponding authors. SCP: The corresponding author is from abroad. MCP: The corresponding author is from their own country; **(C)** Co-cited authors cooperation network map. From: VOSviewer.

Among the 4076 publications related to oxidative stress in DN, a total of 19,372 authors contributed. The top ten authors by publication count are listed in **Table 4**. The five authors with the highest number of publications are: Sun Lin (n=33), Li Ping (n=29), Cooper, Mark E (n=27), Shi Yonghong (n=24), and Li Yan (n=24). A visualization map was generated for authors who published more than 10 articles (**Figure 7A**). Sun Lin has the largest node, indicating that he published the most related articles. The collaboration between authors is evident, with notable partnerships such as between Sun Lin and Xiao Li, and Wu Meiming and Shi Yonghong. **Figure 7B** shows the country distribution of corresponding authors.

**Table 4:** Top 10 authors and co-cited authors with the highest number of articles.

Rank	Author	Documents	Co-Cited Author	Count
1	Sun Lin	33	Forbes, JM	669
2	Li Ping	29	Jha, JC	508
3	Cooper, Mark E	27	Brownlee, M	501
4	Shi Yonghong	24	Wang Y	416
5	Li Yan	24	Sharma, K	402
6	Liu Yan	21	Kitada, M	382
7	Zhang Wei	21	Vallon, V	374
8	Li Ying	20	Navarro-González, JF	374
9	Wang Li	20	Alicic, RZ	332
10	Sahebkar, Amirhossein	20	Kanwar, YS	316

#### 3.4.2 Co-cited authors

Among 83,410 co-cited authors, the most frequently co-cited author is Forbes, JM (n=669), followed by Jha, JC (n=508), Brownlee, M (n=501), Wang, Y (n=416), Sharma, K (n=402), Kitada, M (n=382), Vallon, V (n=374), Navarro-González, JF (n=374), Alicic, RZ (n=332), and Kanwar, YS (n=316), as shown in **Table 4**. A visualization network map was created for co-cited authors with more than 300 co-citations (**Figure 7C**). The node representing Forbes, JM is the largest, reflecting his position as the most co-cited author. The network also highlights close collaborations among several co-cited authors, such as Kitada, M and Coughlan, ML, and Jha, JC and Gorin, Y, indicating frequent communication and collaboration among these researchers.

### 3.5 Keywords

**Figure 8:**
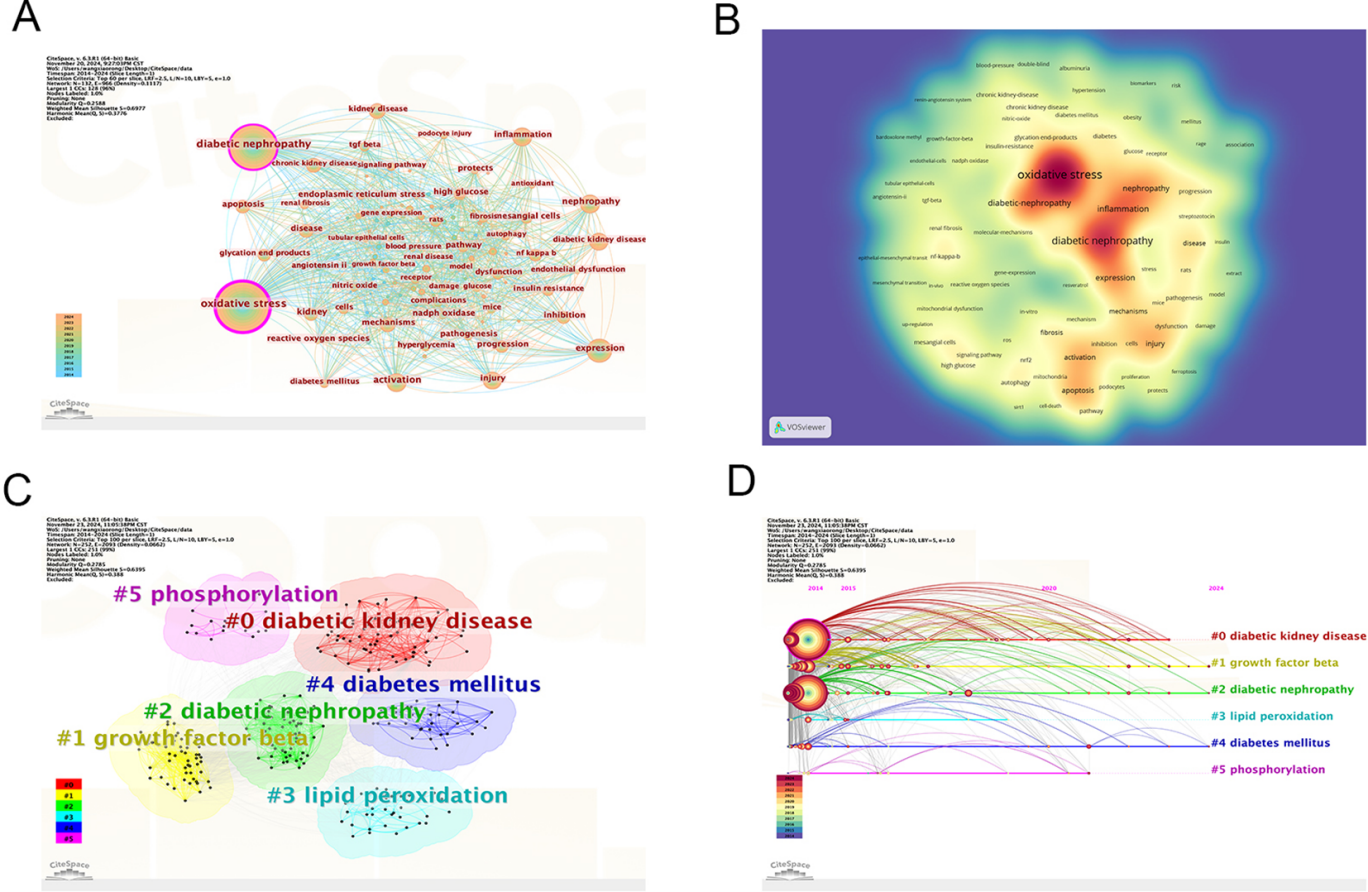
Bibliographic analysis of keywords. **(A)** Visualization of keywords; **(B)** Density map of keyword cooperation network; **(C)** Clustering map of keyword topics; **(D)** Timeline view of keywords. From: CiteSpace.

**Table 5** lists the ten most frequently occurring keywords in the analysis: oxidative stress (n=3683), diabetic nephropathy (n=2338), expression (n=715), nephropathy (n=549), activation (n=530), inflammation (n=480), injury (n=480), kidney disease (n=411), mechanisms (n=390), and chronic kidney disease (n=467). In **Figure 8A**, oxidative stress and DN are represented as the largest nodes. The density map of the keyword co-occurrence network shows that oxidative stress and DN are positioned at the center (**Figure 8B**). The centrality of keywords also reflects the research frontiers; keywords with strong centrality include oxidative stress, DN, and activation. All keywords were clustered, and six types of labels were calculated (**Figure 8C**). These clusters include diabetic kidney disease (DKD), growth factor beta, DN, lipid peroxidation, diabetes mellitus, and phosphorylation. **Figure 8D** shows the timeline of keyword cluster changes.

**Table 5:** The 10 keywords with the highest frequency.

Rank	Keyword	Frequency	Centrality
1	Oxidative stress	2683	0.43
2	Diabetic nephropathy	2338	0.23
3	Expression	715	0.04
4	Nephropathy	549	0.05
5	Activation	530	0.06
6	Inflammation	480	0.05
7	Injury	480	0.02
8	Kidney disease	411	0.05
9	Mechanisms	369	0.03
10	Chronic kidney disease	362	0.04

**Figure 9:**
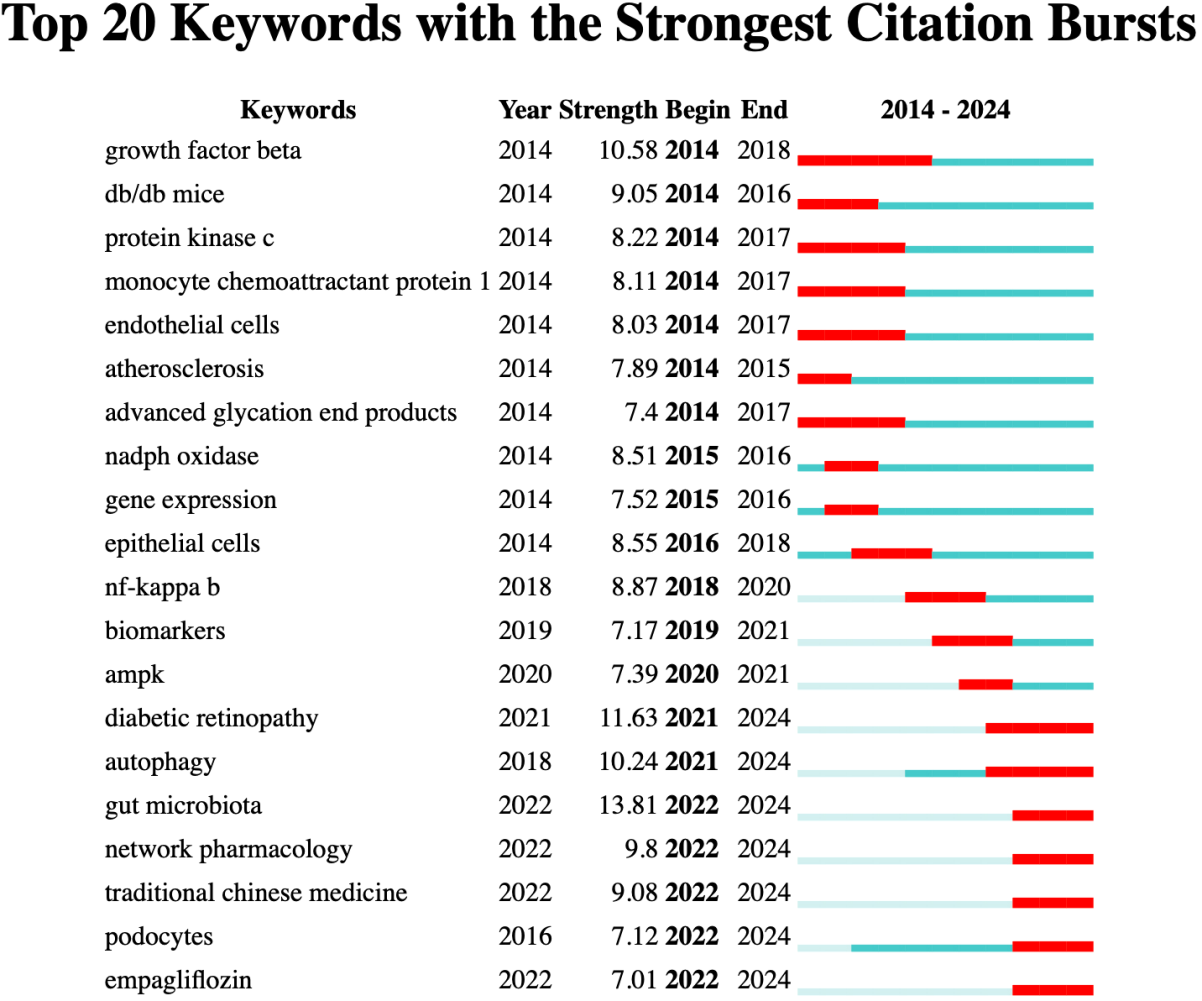
Top 20 keywords with the strongest citation bursts.

According to the burst detection analysis (**Figure 9**), the strongest keyword burst is “gut microbiota” (13.81), which has emerged as a recent hot topic.

### 3.6 Analysis of references

**Figure 10:**
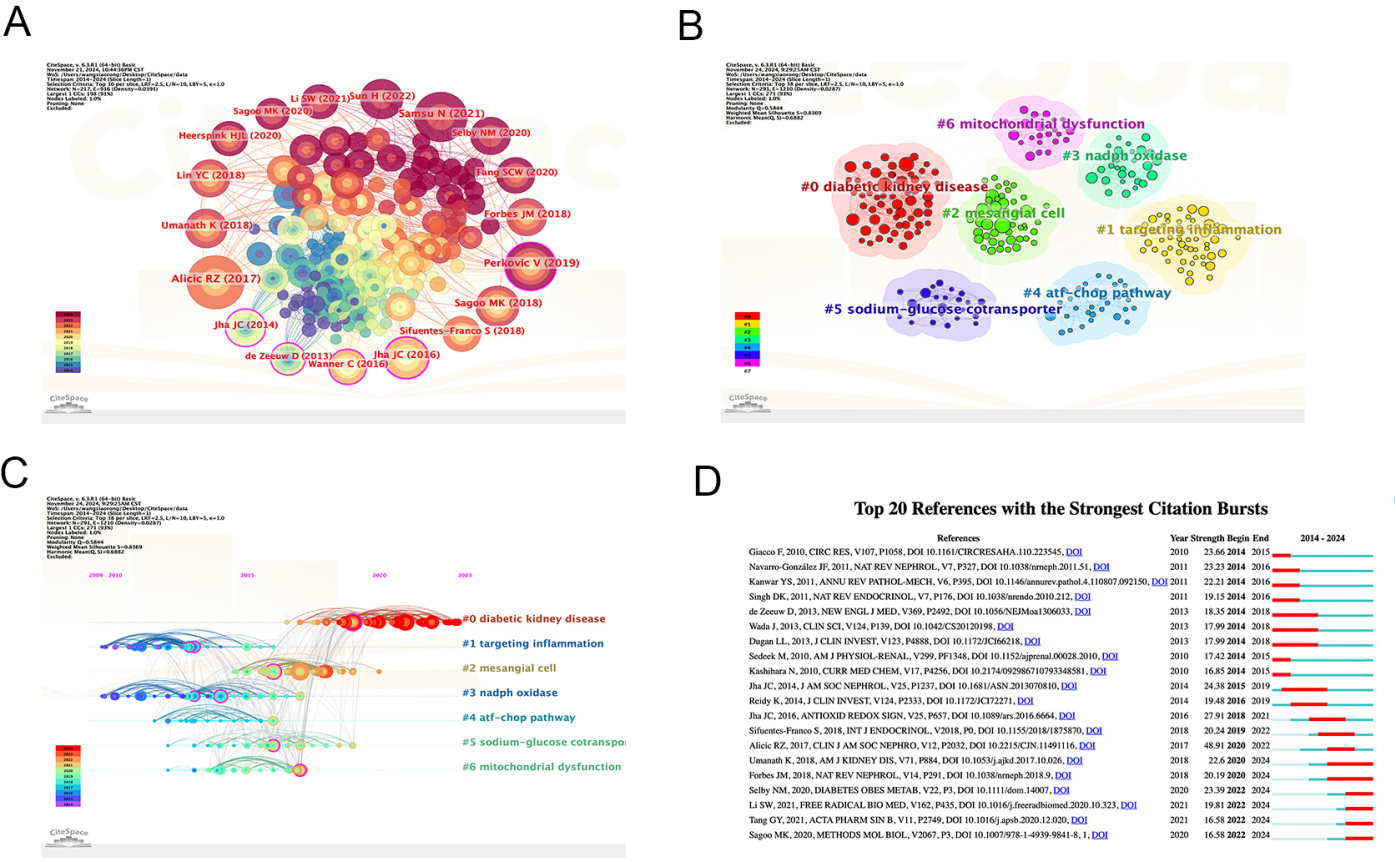
Bibliographic analysis of cited references. **(A)** Map of cited references; **(B)** Clustering map of cited reference topics; **(C)** Timeline view of references; **(D)** The top 20 references with the strongest citation bursts in research. From: CiteSpace.

CiteSpace was used to perform cluster analysis and visualization of co-cited references. The clustering analysis of co-cited references effectively presents the research themes and emerging hotspots. The top ten most cited references are listed in **Table 6**, with the most frequently cited being Alicic RZ, 2017 (n=144). Other highly cited articles include Samasu, 2021 (n=126), Perkovic V, 2019 (n=117), Sagoo MK, 2018 (n=91), Jha JC, 2016 (n=86), and Umanath K, 2018 (n=82). The map of cited references shows that the larger the circle, the more frequent the citation (**Figure 10A**). In the cluster analysis (**Figure 10B**), seven clusters were identified, labeled as follows: #0 DKD, #1 Targeting Inflammation, #2 Mesangial Cell, #3 NADPH Oxidase, #4 ATF-CHOP Pathway, #5 Sodium-Glucose Cotransporter, and #6 Mitochondrial Dysfunction. These findings suggest that the current research hotspots in this field focus on therapeutic prospects and extracellular matrix accumulation. The timeline of reference clusters (**Figure 10C**) illustrates the evolution of these clusters over time. In the burst detection analysis of co-cited references (**Figure 10D**), the most significant citation burst was observed for Alicic RZ, 2017 (48.91). Recent citation bursts were observed for the following articles: Umanath K, 2018 (22.6), Forbes JM, 2018 (20.19), Selby NM, 2020 (23.39), Li SW, 2021 (19.81), Tang GY, 2021 (16.58), and Sagoo MK, 2020 (16.58).

**Table 6:** Top 10 highly cited literature.

Rank	Document	Citations	Source	Centrality
1	Alicic RZ (2017)	144	CLIN J AM SOC NEPHRO	0.05
2	Samasu(2021)	126	BIOMED RES INT	0.04
3	Perkovic V (2019)	117	NEW ENGL J MED	0.14
4	Sagoo MK (2018)	91	FREE RADICAL BIO MED	0.05
5	Umanath K (2018)	86	AM J KIDNEY DIS	0.21
6	Jha JC (2016)	84	ANTIOXID REDOX SIGN	0.08
7	Sun H (2022)	82	DIABETES RES CLIN PR	0.08
8	Lin YC (2018)	77	J FORMOS MED ASSOC	0.08
9	Forbes JM (2018) J	77	NAT REV NEPHROL	0.04
10	ha JC (2014)	73	J AM SOC NEPHROL	0.14

## 4 Discussion

This is the first study to explore the structural correlations and temporal dynamics of DN pathogenesis from the perspective of oxidative stress. Using VOSviewer, the Bibliometrix R package, and CiteSpace, we conducted a comprehensive analysis of publications in this field since 2014, producing visual representations, such as quantitative data on authors, journals, countries/regions, keywords, and references. These results provide valuable insights into the research hotspots and trends in this area.

### 4.1 Overview

As more individuals with poorly controlled DM develop DN (30, 31), the global focus on understanding the mechanisms of DN has intensified. As a result, the number of publications related to oxidative stress in DN has steadily increased over the past decade, with an average of 408 publications per year. This growth highlights oxidative stress as one of the key pathogenic mechanisms in DN, attracting increasing attention from researchers.

China and the United States lead the research on oxidative stress in DN, supported by their strong economies, which provide significant funding for such studies. Other Asian countries, such as India, Japan, and Iran, have also made notable contributions, likely due to the higher incidence of DN in these populations (32). The top five institutions contributing to this research are all located in China, which may be linked to China’s high DM prevalence and large population of DM patients (33). Although Chinese institutions dominate the academic output in oxidative stress research, their distribution is uneven, with most concentrated in economically developed cities and limited collaboration between them.

Regarding authors, Sun Lin is the leading contributor, with 33 publications. He is affiliated with the Department of Nephrology at Xiangya Second Hospital of Central South University in Hunan, China. His latest study, published in October 2023, investigates how DsbA-L interacts with catalase in peroxisomes to improve tubular oxidative damage in DN. His team demonstrated that DsbA-L can enhance the activity and content of catalase, thereby enriching our understanding of the mechanisms underlying renal tubular injury in DN (34). Additionally, in April 2023, Sun Lin’s team explored mitochondrial oxidative stress and suggested that mitochondrial-activated transcription factor 5 (ATF5) may serve as a potential target for DN prevention (35). Forbes, JM, based at the University of Queensland in Brisbane, Australia, is the most co-cited author in this field, underscoring his authoritative role in this area. However, both Sun Lin and Forbes JM primarily collaborate with domestic scholars in the same field, with limited international cooperation. This lack of global collaboration is a common issue in the field. We believe that international collaboration within the same research domain could foster complementary strengths and should be encouraged to deepen our understanding of DN pathogenesis.

When exploring oxidative stress in DN, key journals, publications, and core authors provide valuable insights. We would first look at high-impact journals with a large number of publications, such as *INTERNATIONAL JOURNAL OF MOLECULAR SCIENCES* (n=117), *FRONTIERS IN PHARMACOLOGY* (n=111), and *BIOMEDICINE* & *PHARMACOTHERAPY* (n=99). Additionally, searching other core journals (**Figure 5B**), beyond just the top ten, would provide broader and more specific information to advance our understanding of the field.

### 4.2 Hotspots and frontiers

Keyword analysis is crucial for identifying research hotspots and trends, as it effectively reflects the core research themes. The most frequent keywords in this study are DN and oxidative stress, which serve as the main axis of the research, with other topics revolving around these two focal points.

In the burst detection analysis, gut microbiota exhibited the strongest burst intensity. Disruption of the gut-kidney axis and microbial metabolites has been reported to exacerbate oxidative stress, accelerating the onset and progression of DN (36). Studies have shown that gut microbiota remodeling, by suppressing oxidative stress and inflammation, can improve DN (37, 38). Other recent emerging keywords include autophagy, as it is known that autophagy and oxidative stress interact to regulate DN kidney injury (39), with more researchers exploring the complex relationship between the two (40). Theodomir et al. pointed out that geniposide enhances ULK1-mediated autophagy to mitigate oxidative stress in DN (41). Keywords with lower burst strength are also worth attention, as they represent emerging research areas. The two keywords with the lowest burst strength are empagliflozin and podocytes. Empagliflozin is a selective SGLT2 inhibitor (42). Related studies have indicated that this drug can improve DN by counteracting hyperglycemia, inhibiting oxidative stress, and reducing inflammation (43). Research by Kelly et al. suggests that empagliflozin may improve DN through both glucose-dependent and independent mechanisms (44). Podocyte injury is a major cause of albuminuria in DN, and oxidative stress plays a crucial role in podocyte damage (45).

Clusters, to some extent, indicate the research frontiers. In the keyword clustering analysis (**Figure 8C**), the identified clusters include DKD, growth factor beta, DN, DM, lipid peroxidation, and phosphorylation. DKD is a broad term that encompasses various functional and structural kidney changes caused by DM, including DN (46). Oxidative stress plays a pivotal role in both DKD and DN, with chronic hyperglycemia acting as a core trigger and mitochondria serving as the primary site of ROS generation (47). Persistent hyperglycemia disrupts mitochondrial function, causing oxidative pathway dysregulation and excessive ROS production, which ultimately induces oxidative stress (48).

The impact of oxidative stress differs across the stages of DKD and DN. In the early stages of DN, although no significant structural damage is observed, oxidative stress triggered by hyperglycemia begins to exert its effects (49, 50). Excessive advanced glycation end-products (AGEs) and polyol pathway flux activate protein kinase C (PKC) and TGF-β signaling pathways, which further amplify ROS production (51, 52). This results in macromolecular damage to proteins, lipids, and DNA in kidney cells (53, 54). At this stage, ROS functions as a secondary messenger, activating multiple signaling pathways, including TGF-β, and inducing their phosphorylation (55). This process accelerates the depletion of cellular antioxidants, ultimately resulting in the onset of oxidative stress (56). At this stage, oxidative stress primarily impairs renal tubular epithelial cell function, weakens the antioxidant defense system, and causes mild kidney dysfunction (57). For instance, lipid peroxidation of podocyte membranes induced by ROS disrupts cellular integrity, leading to podocyte loss and damage to the glomerular filtration barrier, resulting in microalbuminuria (58). Additionally, ROS generated by oxidative stress in mesangial cells promote extracellular matrix (ECM) deposition, laying the foundation for advanced kidney fibrosis (59, 60).

As DN progresses to later stages, structural damage becomes more pronounced (61), and the effects of oxidative stress intensify (62). Oxidative stress not only exacerbates damage to renal tubular and glomerular endothelial cells but also accelerates renal fibrosis (63). ROS promote tubulointerstitial damage and activate fibroblasts, leading to interstitial fibrosis and eventually causing severe structural and functional kidney impairment (64). Specifically, oxidative stress disrupts the tight junctions of glomerular endothelial cells, increasing vascular permeability and allowing large molecules, such as proteins, to leak into the urine, thereby worsening proteinuria. Additionally, oxidative stress alters hemodynamics, further aggravating glomerular injury (65). As tubulointerstitial damage and fibrosis advance, the role of oxidative stress in late-stage DN becomes increasingly pronounced (66).

Co-cited references represent the background and baseline of the research field. The most frequently cited reference is Alicic RZ, 2017, which systematically investigates the risk factors and pathophysiological changes of DN (9). This study exhibits the strongest citation burst and lays a solid theoretical foundation for further research on DN. In the reference cluster analysis, Cluster 1 focuses on targeting inflammation, with oxidative stress being one of the main drivers of inflammatory responses (67). Excessive ROS activate inflammatory signaling pathways such as NF-κB and MAPK, promoting the release of inflammatory mediators and exacerbating renal injury (11, 68). Meanwhile, inflammatory factors such as TNF-α and IL-1β activate NADPH oxidase, leading to increased ROS production and forming a positive feedback loop between oxidative damage and inflammation, thereby accelerating DN progression (69). Cluster 3 focuses on NADPH oxidase, the only known enzyme family responsible for ROS production, which includes seven isoforms: Nox1, Nox2, Nox3, Nox4, Nox5, DUOX1, and DUOX2 (70–72). Among these, Nox4 is a major research target and has been extensively studied (73). Mitochondrial dysfunction (#6) is characterized by excessive ROS production within the mitochondria, a reduction in ATP, mutations in mitochondrial DNA, and subsequent mitochondrial swelling and rupture (74). Excessive ROS modulate the phosphorylation of Bcl-2 proteins, which induces changes in mitochondrial membrane permeability, ultimately leading to mitochondrial dysfunction (75). Reference clusters offer valuable guidance for future research directions.

In recent years, inhibiting oxidative stress has become a major therapeutic strategy for DN. The antioxidant bioactivity of medicinal plants in DN treatment warrants attention. Studies have shown that mushrooms collected from Turkey exhibit strong antioxidant potential (76). The round-stemmed mushroom from safe areas has also been confirmed as a good source of antioxidants (77). Bahare et al. reported that Achillea spp. demonstrates powerful antioxidant effects in both cell and animal models (78). Propolis, a natural remedy, has also shown potential in improving kidney function by mitigating oxidative damage (79). Other studies have explored novel synthetic organic selenium compounds that exhibit antioxidant effects on rat kidneys (80). Hydrogen sulfide has also been shown to protect kidneys by reducing ROS and inhibiting oxidative stress (81, 82). Metformin and angiotensin receptor blockers (ARBs) have also been found to reduce oxidative stress markers and enhance antioxidant enzyme activity in the kidney, providing therapeutic benefits for DN (83, 84). Traditional Chinese Medicine (TCM) has significantly contributed to the treatment of DN. 6-Shogaol, a major active component of ginger, inhibits oxidative stress and restores Nrf2 expression in the kidneys, thereby improving DN (85). Mao et al. demonstrated that Huangkui capsules protect the kidneys by controlling MDA, SOD, and Nox4 expression in DN (86). Tongxinluo, a novel Chinese herbal compound, significantly reduces Nox4 expression in DN rats, inhibits oxidative stress, and decreases podocyte apoptosis (87). Zuogui Wan has been shown to protect kidneys by reducing oxidative stress and inhibiting apoptosis in DN (88).

## 5 Strengths and limitations

In comparison to traditional reviews, this study utilizes statistical techniques to analyze 4076 publications, providing a more intuitive and comprehensive reflection of the research hotspots and development trends in this field from 2014 to the present. The results provide valuable insights for researchers. However, this approach has some limitations. Although the WoSCC database is one of the most commonly used and authoritative databases, its data resources represent only a specific scope of a particular field. Additionally, this study analyzed only English-language publications, which may have excluded important articles published in other languages. Finally, the existence of synonyms could introduce bias into the results. To mitigate these issues, the search strategy must be used with caution. Nonetheless, from a bibliometric perspective, we believe that our results accurately reflect the research on oxidative stress related to DN since 2014.

## 6 Conclusion

In conclusion, since 2014, publications focusing on the role of oxidative stress in DN have steadily increased, underscoring its pivotal role in DN pathogenesis. Oxidative stress drives kidney damage through mechanisms like lipid peroxidation, mitochondrial dysfunction, and inflammation, making it a central target for therapeutic research. This study provides critical insights for basic scientists to identify research trends, prioritize key areas, and facilitate future investigations into molecular mechanisms and the development of innovative antioxidant therapies. A deeper understanding of these processes is essential for advancing treatment strategies and improving clinical outcomes in DN patients.

## Data Availability

The original contributions presented in the study are included in the article/supplementary material. Further inquiries can be directed to the corresponding authors.
